# Adverse Event Signal Detection Using Patients’ Concerns in Pharmaceutical Care Records: Evaluation of Deep Learning Models

**DOI:** 10.2196/55794

**Published:** 2024-04-16

**Authors:** Satoshi Nishioka, Satoshi Watabe, Yuki Yanagisawa, Kyoko Sayama, Hayato Kizaki, Shungo Imai, Mitsuhiro Someya, Ryoo Taniguchi, Shuntaro Yada, Eiji Aramaki, Satoko Hori

**Affiliations:** 1 Division of Drug Informatics Keio University Faculty of Pharmacy Tokyo Japan; 2 Nakajima Pharmacy Hokkaido Japan; 3 Nara Institute of Science and Technology Nara Japan

**Keywords:** cancer, anticancer drug, adverse event, side effect, patient-reported outcome, patients’ voice, patient-oriented, patient narrative, natural language processing, deep learning, pharmaceutical care record, SOAP

## Abstract

**Background:**

Early detection of adverse events and their management are crucial to improving anticancer treatment outcomes, and listening to patients’ subjective opinions (patients’ voices) can make a major contribution to improving safety management. Recent progress in deep learning technologies has enabled various new approaches for the evaluation of safety-related events based on patient-generated text data, but few studies have focused on the improvement of real-time safety monitoring for individual patients. In addition, no study has yet been performed to validate deep learning models for screening patients’ narratives for clinically important adverse event signals that require medical intervention. In our previous work, novel deep learning models have been developed to detect adverse event signals for hand-foot syndrome or adverse events limiting patients’ daily lives from the authored narratives of patients with cancer, aiming ultimately to use them as safety monitoring support tools for individual patients.

**Objective:**

This study was designed to evaluate whether our deep learning models can screen clinically important adverse event signals that require intervention by health care professionals. The applicability of our deep learning models to data on patients’ concerns at pharmacies was also assessed.

**Methods:**

Pharmaceutical care records at community pharmacies were used for the evaluation of our deep learning models. The records followed the SOAP format, consisting of subjective (S), objective (O), assessment (A), and plan (P) columns. Because of the unique combination of patients’ concerns in the S column and the professional records of the pharmacists, this was considered a suitable data for the present purpose. Our deep learning models were applied to the S records of patients with cancer, and the extracted adverse event signals were assessed in relation to medical actions and prescribed drugs.

**Results:**

From 30,784 S records of 2479 patients with at least 1 prescription of anticancer drugs, our deep learning models extracted true adverse event signals with more than 80% accuracy for both hand-foot syndrome (n=152, 91%) and adverse events limiting patients’ daily lives (n=157, 80.1%). The deep learning models were also able to screen adverse event signals that require medical intervention by health care providers. The extracted adverse event signals could reflect the side effects of anticancer drugs used by the patients based on analysis of prescribed anticancer drugs. “Pain or numbness” (n=57, 36.3%), “fever” (n=46, 29.3%), and “nausea” (n=40, 25.5%) were common symptoms out of the true adverse event signals identified by the model for adverse events limiting patients’ daily lives.

**Conclusions:**

Our deep learning models were able to screen clinically important adverse event signals that require intervention for symptoms. It was also confirmed that these deep learning models could be applied to patients’ subjective information recorded in pharmaceutical care records accumulated during pharmacists’ daily work.

## Introduction

Increasing numbers of people are expected to develop cancers in our aging society [1-3]. Thus, there is increasing interest in how to detect and manage the side effects of anticancer therapies in order to improve treatment regimens and patients’ quality of life [4-8]. The primary approaches for side effect management are “early signal detection and early intervention” [9-11]. Thus, more efficient approaches for this purpose are needed.

It has been recognized that patients’ voices concerning adverse events represent an important source of information. Several studies have indicated that the number, severity, and time of occurrence of adverse events might be underevaluated by physicians [12-15]. Thus, patient-reported outcomes (PROs) have recently received more attention in the drug evaluation process, reflecting patients’ real voices. Various kinds of PRO measures have been developed and investigated in different disease populations [16,17]. Health care authorities have also encouraged the pharmaceutical industry to use PROs for drug evaluation [18,19], and it is becoming more common to take PRO assessment results into consideration for drug marketing approval [20,21]. Similar trends can be seen in the clinical management of individual patients. Thus, health care professionals have an interest in understanding how to appropriately gather patients’ concerns in order to improve safety management and clinical decisions [22-24].

The applications of deep learning for natural language processing have expanded dramatically in recent years [25]. Since the development of a high-performance deep learning model in 2018 [26], attempts to apply cutting-edge deep learning models to various kinds of patient-generated text data for the evaluation of safety events or the analysis of unscalable subjective information from patients have been accelerating [27-31]. Most studies have been conducted to use patients’ narrative data for pharmacovigilance [27,32-35], while few have been aimed at improvement of real-time safety monitoring for individual patients. In addition, there have been some studies on adverse event severity grading based on health care records [36-39], but none has yet aimed to extract clinically important adverse event signals that require medical intervention from patients’ narratives. It is important to know whether deep learning models could contribute to the detection of such important adverse event signals from concern texts generated by individual patients.

To address this question, we have developed deep learning models to detect adverse event signals from individual patients with cancer based on patients’ blog articles in online communities, following other types of natural language processing–related previous work [40,41]. One deep learning model focused on the specific symptom of hand-foot syndrome (HFS), which is one of the typical side effects of anticancer treatments [42], and another focused on a broad range of adverse events that impact patients’ activities of daily living [43]. We showed that our models can provide good performance scores in targeting adverse event signals. However, the evaluation relied on patients’ narratives from the patients’ blog data used for deep learning model training, so further evaluation is needed to ensure the validity and applicability of the models to other texts regarding patients’ concerns. In addition, the blog data source did not contain medical information, so it was not feasible to assess whether the models could contribute to the extraction of clinically important adverse event signals.

To address these challenges, we focused on pharmaceutical care records written by pharmacists at community pharmacies. The gold standard format for pharmaceutical care records in Japan is the SOAP (subjective, objective, assessment, plan)-based document that follows the “problem-oriented system” concept proposed by Weed [44] in 1968. Pharmacists track patients’ subjective concerns in the S column, provide objective information or observations in the O column, give their assessment from the pharmacist perspective in the A column, and suggest a plan for moving forward in the P column [45,46]. We considered that SOAP-based pharmaceutical care records could be a unique data source suitable for further evaluation of our deep learning models because they contain both patients’ concerns and professional health care records by pharmacists, including the medication prescription history with time stamps. Therefore, this study was designed to assess whether our deep learning models could extract clinically important adverse event signals that require intervention by medical professionals from these records. We also aimed to evaluate the characteristics of the models when applied to patients’ subjective information noted in the pharmaceutical care records, as there have been only a few studies on the application of deep learning models to patients’ concerns recorded during pharmacists’ daily work [47-49].

Here, we report the results of applying our deep learning models to patients’ concern text data in pharmaceutical care records, focusing on patients receiving anticancer treatment.

## Methods

### Data Source

The original data source was 2,276,494 pharmaceutical care records for 303,179 patients, created from April 2020 to December 2021 at community pharmacies belonging to the Nakajima Pharmacy Group in Japan [50]. To focus on patients with cancer, records of patients with at least 1 prescription for an anticancer drug were retrieved by sorting individual drug codes (YJ codes) used in Japan (YJ codes starting with 42 refer to anticancer drugs). Records in the S column (ie, S records) were collected from the patients with cancer as the text data of patients’ concerns for deep learning model analysis.

### Deep Learning Models

The deep learning models used for this research were those that we constructed based on patients’ narratives in blog articles posted in an online community and that showed the best performance score in each task in our previous work (ie, a Bidirectional Encoder Representations From Transformers [BERT]–based model for HFS signal extraction [42] and a T5-based model for adverse event signal extraction [43]). BERT [26] and T5 [51] both belong to a type of deep learning model that has recently shown high performance in several studies [29,52]. Hereafter, we refer to the deep learning model for HFS signals as the HFS model, the model for any adverse event signals as All AE (ie, all or any adverse events) model, and the model for adverse event signals limited to patients’ activities of daily living as the AE-L (adverse events limiting patients’ daily lives) model. It was also confirmed that these deep learning models showed similar or higher performance scores for the HFS, All AE, or AE-L identification tasks using 1000 S records randomly extracted from the data source of this study compared to the values obtained in our previous work [42,43] (the performance scores of sentence-level tasks from our previous work are comparable, as the mean number of words in the sentences in the data source in our previous work was 32.7 [SD 33.9], which is close to that of the S records used in this study, 38.8 [SD 29.4]). The method and results of the performance-level check are described in detail in Multimedia Appendix 1 [42,43]. We applied the deep learning models to all text data in this study without any adjustment in setting parameters from those used in constructing them based on patient-authored texts in our previous work [42,43].

### Evaluation of Extracted S Records by the Deep Learning Models

In this study, we focused on the evaluation of S records that our deep learning models extracted as HFS or AE-L positive. Each positive S record was assessed as if it was a true adverse event signal, a sort of adverse event symptom, whether or not an intervention was made by health care professionals. We also investigated the kind of anticancer treatment prescription in connection with each adverse event signal identified in S records.

To assess whether an extracted positive S record was a true adverse event signal, we used the same annotation guidelines as in our previous work [43]. In brief, each S record was treated as an “adverse event signal” if any untoward medical occurrence happened to the patient, regardless of the cause. For the AE-L model only, if a positive S record was confirmed as an adverse event signal, it was further categorized into 1 or more of the following adverse event symptoms: “fatigue,” “nausea,” “vomiting,” “diarrhea,” “constipation,” “appetite loss,” “pain or numbness,” “rash or itchy,” “hair loss,” “menstrual irregularity,” “fever,” “taste disorder,” “dizziness,” “sleep disorder,” “edema,” or “others.”

For the assessment of interventions by health care professionals and anticancer treatment prescriptions, information from the O, A, and P columns and drug prescription history in the data source were investigated for the extracted positive S records. The interventions by health care professionals were categorized in any of the following: “adding symptomatic treatment for the adverse event signal,” “dose reduction or discontinuation of causative anticancer treatment,” “consultation with physician,” “others,” or “no intervention (ie, just following up the adverse event signal).” The actions categorized in “others” were further evaluated individually. For this assessment, we also randomly extracted 200 S records and evaluated them in the same way for comparison with the results from the deep learning model. Prescription history of anticancer treatment was analyzed by primary category of mechanism of action (MoA) with subcategories if applicable (eg, target molecule for kinase inhibitors).

### Applicability Check to Other Text Data Including Patients’ Concerns

To check the applicability of our deep learning models to data from a different source, interview transcripts from patients with cancer were also evaluated. The interview transcripts were created by the Database of Individual Patient Experiences-Japan (DIPEx-Japan) [53]. DIPEx-Japan divides the interview transcripts into sections for each topic, such as “onset of disease” and “treatment,” and posts the processed texts on its website. Processing is conducted by accredited researchers based on qualitative research methods established by the University of Oxford [54]. In this study, interview text data created from interviews with 52 patients with breast cancer conducted from January 2008 to October 2018 were used to assess whether our deep learning models can extract adverse event signals from this source. In total, 508 interview transcripts were included with the approval of DIPEx-Japan.

### Ethical Considerations

This study was conducted with anonymized data following approval by the ethics committee of the Keio University Faculty of Pharmacy (210914-1 and 230217-1) and in accordance with relevant guidelines and regulations and the Declaration of Helsinki. Informed consent specific to this study was waived due to the retrospective observational design of the study with the approval of the ethics committee of the Keio University Faculty of Pharmacy. To respect the will of each individual stakeholder, however, we provided patients and pharmacists of the pharmacy group with an opportunity to refuse the sharing of their pharmaceutical care records by posting an overview of this study at each pharmacy store or on their web page regarding the analysis using pharmaceutical care records. Interview transcripts from DIPEx-Japan were provided through a data sharing arrangement for using narrative data for research and education. Consent for interview transcription and its sharing from DIPEx-Japan was obtained from the participants when the interviews were recorded.

## Results

### Data Set

From the original data source of 2,180,902 pharmaceutical care records for 291,150 patients, S records written by pharmacists for patients with a history of at least 1 prescription of an anticancer drug were extracted. This yielded 30,784 S records for 2479 patients with cancer (Table 1). The mean and median number of words in the S records were 38.8 (SD 29.4) and 32 (IQR 20-50), respectively. We applied our deep learning models, HFS, All AE, and AE-L, to these 30,784 S records for the evaluation of the deep learning models for adverse event signal detection.

For interview transcripts created by DIPEx-Japan, the mean and median number of words were 428.9 (SD 160.9) and 416 (IQR 308-526), respectively, in the 508 transcripts for 52 patients with breast cancer.

**Table 1:** Characteristics of the pharmaceutical care records.

Item		Values
Duration		April 2020 to December 2021 (1 year and 9 months)
**Patients**		
	Total, n	291,150
	At least 1 anticancer drug prescription, n (%)	2479 (0.9)
**Pharmaceutical care records**		
	SOAP^a^ records for all patients, n	2,180,902
	S records for patients with at least 1 anticancer drug prescription, n (%)	30,784 (1.4)
**Words in S^b^ records for patients with at least 1 anticancer drug prescription**		
	Mean (SD)	38.8 (29.4)
	Median (IQR)	32 (20-50)

^a^SOAP: subjective, objective, assessment, plan.

^b^S: subjective.

### Application of the HFS Model

#### Overview

First, we applied the HFS model to the S records for patients with cancer. The BERT-based model was used for this research as it showed the best performance score in our previous work [42].

#### S Records Extracted as HFS Positive

The S records extracted as HFS positive by the HFS model (Table 2) amounted to 167 (0.5%) records for 119 (4.8%) patients. A majority of the patients had 1 HFS-positive record in their S records (n=91, 76.5%), while 2 patients had as many as 6 (1.7%) HFS-positive records. When we examined whether the extracted S records were true adverse event signals or not, 152 records were confirmed to be adverse event signals, while the other 15 records were false-positives. All the false-positive S records were descriptions about the absence of symptoms or confirmation of improving condition (eg, “no diarrhea, mouth ulcers, or limb pain so far” or “the skin on the soles of my feet has calmed down a lot with this ointment”). Some examples of S records that were predicted as HFS positive by the model are shown in Table S1 in Multimedia Appendix 2.

The same examination was conducted with interview transcripts from DIPEx-Japan. Only 1 (0.2%) transcript was extracted as HFS positive by the HFS model, and it was a true adverse event signal (100%). The actual transcript extracted as HFS positive is shown in Table S2 in Multimedia Appendix 2.

**Table 2:** S^a^ records extracted as HFS^b^ positive by the HFS model.

Item			Values
Positive patients (n=2479), n (%)			119 (4.8)
**Positive S records (n=30,784), n (%)**			167 (0.5)
	Positive S records per patient, mean (SD)	1.40 (0.92)	
**Breakdown of patient numbers by number of positive S records, n (%)**			
	1 record	91 (76.5)	
	2 records	18 (15.1)	
	3 records	4 (3.4)	
	4 records	4 (3.4)	
	6 records	2 (1.7)	
**Adverse event signal, n (%)**			
	Yes	152 (91)	
	No (ie, false-positive^c^)	15 (9)	

^a^S: subjective.

^b^HFS: hand-foot syndrome.

^c^All false-positive S records were denial of symptoms or confirmation of improving condition.

#### Interventions by Health Care Professionals

**Figure 1:**
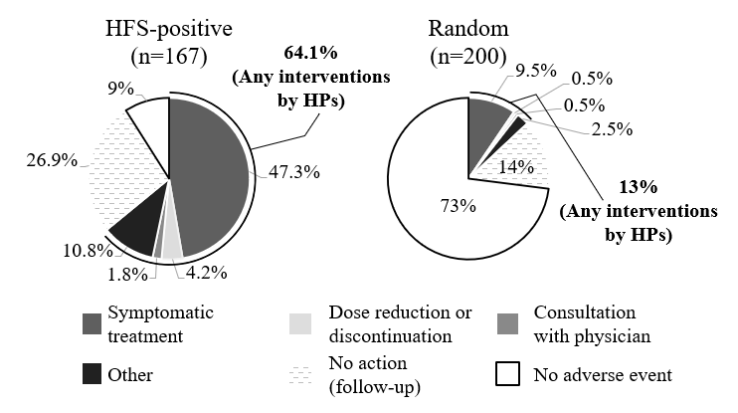
Interventions in cases with HFS-positive S records and in random cases. HFS: hand-foot syndrome; HP: health care professional.

The 167 S records extracted as HFS positive as well as 200 randomly selected records were checked for interventions by health care professionals (Figure 1). The proportion showing any action by health care professionals was 64.1% for 167 HFS-positive S records compared to 13% for the 200 random S records. Among the actions taken for HFS positives, “adding symptomatic treatment” was the most common, accounting for around half (n=79, 47.3%), followed by “other” (n=18, 10.8%). Most “other” actions were educational guidance from pharmacists, such as instructions on moisturizing, nail care, or application of ointment and advice on daily living (eg, “avoid tight socks”).

#### Anticancer Drugs Prescribed

The types of anticancer drugs prescribed for HFS-positive patients are summarized based on the prescription histories in Table 3. For the 152 adverse event signals identified by the HFS model in the previous section, the most common MoA class of anticancer drugs used for the patients was antimetabolite (n=62, 40.8%), specifically fluoropyrimidines (n=59, 38.8%). Kinase inhibitors were next (n=49, 32.2%), with epidermal growth factor receptor (EGFR) inhibitors and multikinase inhibitors as major subgroups (n=28, 18.4% and n=14, 9.2%, respectively). The third and fourth most common MoAs were aromatase inhibitors (n=24, 15.8%) and antiandrogen or estrogen drugs (n=7, 4.6% each) for hormone therapy.

**Table 3:** MoA classes of anticancer drugs prescribed for patients with adverse event signals identified by the HFS model (n=152).

Anticancer drugs		Adverse event signals identified by HFS model, n (%)
**Antimetabolites**		
	Overall	62 (40.8)
	Fluoropyrimidine	59 (38.8)
	Folate analog	3 (2)
**Kinase inhibitors**		
	Overall	49 (32.2)
	EGFR^a^	28 (18.4)
	Multi	14 (9.2)
	VEGF^b^	6 (3.9)
	HER2^c^	1 (0.7)
Aromatase inhibitors		24 (15.8)
Antiandrogens		7 (4.6)
Antiestrogens		7 (4.6)
CDK4/6^d^ inhibitors		3 (2)
Alkylating agents		1 (0.7)

^a^EGFR: epidermal growth factor receptor.

^b^VEGF: vascular endothelial growth factor.

^c^HER2: human epidermal growth factor receptor-2.

^d^CDK4/6: cyclin-dependent kinase 4/6.

### Application of the All AE or AE-L model

#### Overview

The All AE and AE-L models were also applied to the same S records for patients with cancer. The T5-based model was used for this research as it gave the best performance score in our previous work [43].

#### S Records Extracted as All AE or AE-L positive

The numbers of S records extracted as positive were 7604 (24.7%) for 1797 patients and 196 (0.6%) for 142 patients for All AE and AE-L, respectively. In the case of All AE, patients tended to have multiple adverse event positives in their S records (n=1315, 73.2% of patients had at least 2 positives). In the case of AE-L, most patients had only 1 AE-L positive (n=104, 73.2%), and the largest number of AE-L positives for 1 patient was 4 (2.8%; Table 4).

We focused on AE-L evaluation due to its greater importance from a medical viewpoint and lower workload for manual assessment, considering the number of positive S records. Of the 197 AE-L–positive S records, it was confirmed that 157 (80.1%) records accurately extracted adverse event signals, while 39 (19.9%) records were false-positives that did not include any adverse event signals (Table 4). The contents of the 39 false-positives were all descriptions about the absence of symptoms or confirmation of improving condition, showing a similar tendency to the HFS false-positives (eg, “The diarrhea has calmed down so far. Symptoms in hands and feet are currently fine” and “No symptoms for the following: upset in stomach, diarrhea, nausea, abdominal pain, abdominal pain or stomach cramps, constipation”). Examples of S records that were predicted as AE-L positive are shown in Table S3 in Multimedia Appendix 2.

The deep learning models were also applied to interview transcripts from DIPEx-Japan in the same manner. The deep learning models identified 84 (16.5%) and 18 (3.5%) transcripts as All AE or AE-L positive, respectively. Of the 84 All AE–positive transcripts, 73 (86.9%) were true adverse event signals. The false-positives of All AE (n=11, 13.1%) were categorized into any of the following 3 types: explanations about the disease or its prognosis, stories when their cancer was discovered, or emotional changes that did not include clear adverse event mentions. With regard to AE-L, all the 18 (100%) positives were true adverse event signals (Table S4 in Multimedia Appendix 2). Examples of actual transcripts extracted as All AE or AE-L positive are shown in Table S5 in Multimedia Appendix 2.

**Table 4:** S^a^ records extracted as positive by the All AE^b^ or AE-L^c^ model.

Item			Values
**All AE positive**			
	Positive patients (n=2479), n (%)		1797 (72.5)
	**Positive S records (n=30,784), n (%)**		7604 (24.7)
		Positive S records per patient, mean (SD)	4.23 (4.17)
	**Breakdown of patient numbers by number of positive S records, n (%)**		
		1 record	482 (26.8)
		2-5 records	849 (47.3)
		6-10 records	337 (18.8)
		11-20 records	114 (6.3)
		>20 records	15 (0.8)
**AE-L positive**			
	Positive patients (n=2479), n (%)		142 (5.7)
	**Positive S records (n=30,784), n (%)**		196 (0.6)
		Positive S records per patient, mean (SD)	1.38 (0.72)
	**Breakdown of patient numbers by number of positive S records, n (%)**		
		1 record	104 (73.2)
		2 records	26 (18.3)
		3 records	8 (5.6)
		4 record**s**	4 (2.8)
	**Adverse event signal, n (%)**		
		Yes	157 (80.1)
		No (ie, false-positive^d^)	39 (19.9)

^a^S: subjective.

^b^All AE: all (or any of) adverse event.

^c^AE-L: adverse events limiting patients’ daily lives.

^d^All false-positive S records were denial of symptoms or confirmation of improving condition.

#### Interventions by Health Care Professionals

**Figure 2:**
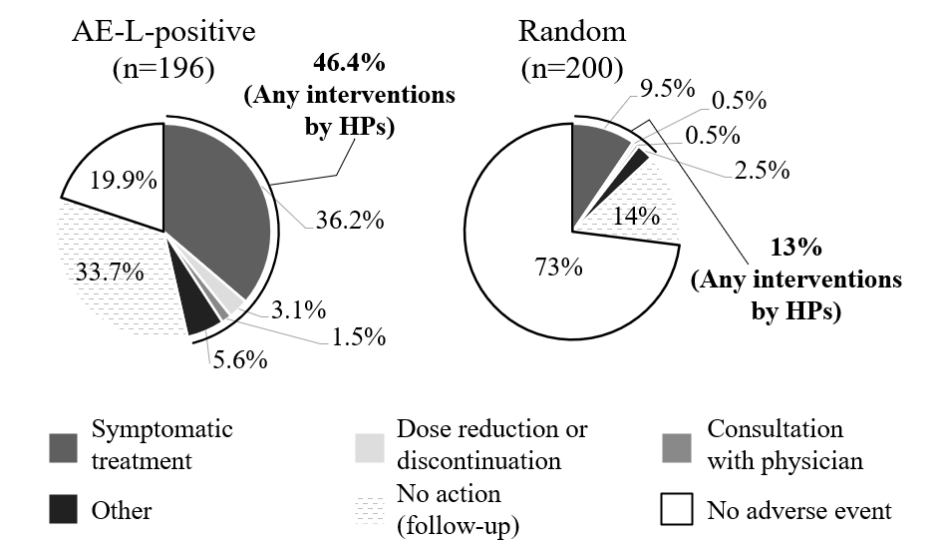
Interventions in cases with AE-L-positive S records and in random cases. AE-L: adverse events limiting patients’ daily lives; HP: health care professional.

Whether or not interventions were made by health care professionals was investigated for the 196 AE-L–positive S records. As in the HFS model evaluation, data from 200 randomly selected S records were used for comparison (Figure 2). In total, 91 (46.4%) records in the 196 AE-L–positive records were accompanied by an intervention, while the corresponding figure in the 200 random records was 26 (13%) records. The most common action in response to adverse event signals identified by the AE-L model was “adding symptomatic treatment” (n=71, 36.2%), followed by “other” (n=11, 5.6%). “Other” included educational guidance from pharmacists, inquiries from pharmacists to physicians, or recommendations for patients to visit a doctor.

#### Anticancer Drugs Prescribed

The types of anticancer drugs prescribed for patients with adverse event signals identified by the AE-L model were summarized based on the prescription histories (Table 5). In connection with the 157 adverse event signals, the most common MoA of the prescribed anticancer drug was antimetabolite (n=62, 39.5%) and fluoropyrimidine (n=53, 33.8%), which accounted for the majority. Kinase inhibitor (n=31, 19.7%) was the next largest category with multikinase inhibitor (n=14, 8.9%) as the major subgroup. These were followed by antiandrogen (n=27, 17.2%), antiestrogen (n=10, 6.4%), and aromatase inhibitor (n=10, 6.4%) for hormone therapy.

**Table 5:** MoA classes of anticancer drugs prescribed for patients with adverse event signals identified by the AE-L model (n=157).

Anticancer drugs		Adverse event signals identified by AE-L model, n (%)	
**Antimetabolites**			
	Overall		62 (39.5)
	Fluoropyrimidine		53 (33.8)
	Trifluridine		5 (3.2)
	Purine analog		3 (1.9)
	Folate analog		1 (0.6)
	Ribonucelotide reductase inhibitor		1 (0.6)
**Kinase inhibitors**			
	Overall		31 (19.7)
	Multi		14 (8.9)
	EGFR^a^		9 (5.7)
	JAK^b^		3 (1.9)
	VEGF^c^		2 (1.3)
	BTK^d^		2 (1.3)
	FLT3^e^		1 (0.6)
Antiandrogens		27 (17.2)	
Antiestrogens		10 (6.4)	
Aromatase inhibitors		10 (6.4)	
Topoisomerase inhibitors		6 (3.8)	
PARP^f^ inhibitors		6 (3.8)	
CDK4/6^g^ inhibitors		4 (2.5)	
Alkylating agents		1 (0.6)	
Anti-CD20^h^ antibodies		1 (0.6)	

^a^EGFR: epidermal growth factor receptor.

^b^JAK: janus kinase.

^c^VEGF: vascular endothelial growth factor.

^c^HER2: human epidermal growth factor receptor-2.

^d^BTK: bruton tyrosine kinase.

^e^FLT3: FMS-like tyrosine kinase-3.

^f^PARP: poly-ADP ribose polymerase.

^g^CDK4/6: cyclin-dependent kinase 4/6.

^h^CD20: cluster of differentiation 20.

#### Adverse Event Symptoms

**Figure 3:**
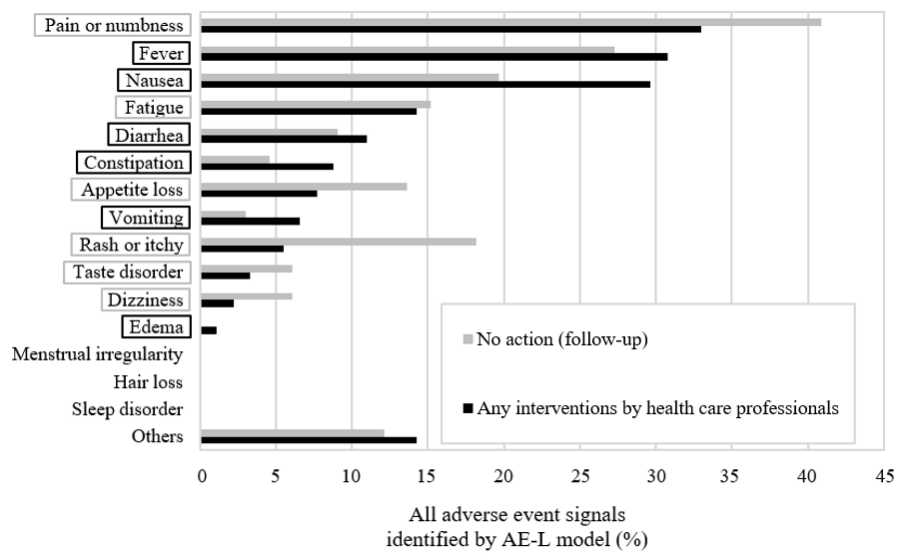
Symptoms of adverse event signals identified by the AE-L model from subjective records by intervention (n=157). AE-L: adverse events limiting patients’ daily lives.

For the 157 adverse event signals identified by the AE-L model, the symptoms were categorized according to the predefined guideline in our previous work [43]. “Pain or numbness” (n=57, 36.3%) accounted for the largest proportion followed by “fever” (n=46, 29.3%) and “nausea” (n=40, 25.5%; Table 6). Symptoms classified as “others” included chills, tinnitus, running tears, dry or peeling skin, and frequent urination. When comparing the proportion of the symptoms associated with or without interventions by health care professionals, a trend toward a greater proportion of interventions was observed in “fever,” “nausea,” “diarrhea,” “constipation,” “vomiting,” and “edema” (Figure 3, black boxes). On the other hand, a smaller proportion was observed in “pain or numbness,” “fatigue,” “appetite loss,” “rash or itchy,” “taste disorder,” and “dizziness” (Figure 3, gray boxes).

**Table 6:** Symptoms of adverse event signals identified by the AE-L model from subjective records (n=157).

Symptoms	Adverse event signals identified by the AE-L model, n (%)
Pain or numbness	57 (36.3)
Fever	46 (29.3)
Nausea	40 (25.5)
Fatigue	23 (14.6)
Rash or itchy	17 (10.8)
Appetite loss	16 (10.2)
Diarrhea	16 (10.2)
Constipation	11 (7)
Vomiting	8 (5.1)
Taste disorder	7 (4.5)
Dizziness	6 (3.8)
Edema	1 (0.6)
Menstrual irregularity	0 (0)
Hair loss	0 (0)
Sleep disorder	0 (0)
Others	21 (13.4)

## Discussion

### Overview

This study was designed to evaluate our deep learning models, previously constructed based on patient-authored texts posted in an online community, by applying them to pharmaceutical care records that contain both patients’ subjective concerns and medical information created by pharmacists. Based on the results, we discuss whether these deep learning models can extract clinically important adverse event signals that require medical intervention, and what characteristics they show when applied to data on patients’ concerns in pharmaceutical care records.

### Performance for Adverse Event Signal Extraction

The first requirement for the deep learning models is to extract adverse event signals from patients’ narratives precisely. In this study, we evaluated the proportion of true adverse event signals in positive S records extracted by the HFS or AE-L model. True adverse event signals amounted to 152 (91%) and 157 (80.1%) for the HFS and AE-L models, respectively (Tables 2 and 4). Given that the proportion of true adverse event signals in 200 randomly extracted S records without deep learning models was 54 (27%; categories other than “no adverse event” in Figures 1 and 2), the HFS and AE-L models were able to concentrate S records with adverse event mentions. Although 15 (9%) for the HFS model and 39 (19.9%) for the AE-L model were false-positives, it was confirmed all of the false-positive records described a lack of symptoms or confirmation of improving condition. We considered that such false-positives are due to the unique feature of pharmaceutical care records, where pharmacists might proactively interview patients about potential side effects of their medications. As the data set of blog articles we used to construct the deep learning models included few such cases (especially comments on lack of symptoms), our models seemed unable to exclude them correctly. Even though we confirmed that the proportion of true “adverse event” signals extracted from the S records by the HFS or AE-L model was more than 80%, the performance scores to extract true “HFS” or “AE-L” signals were not so high based on the performance check using 1000 randomly extracted S records (*F*_1_-scores were 0.50 and 0.22 for true HFS and AE-L signals, respectively; Table S1 in Multimedia Appendix 1). It is considered that the performance to extract true HFS and AE-L signals was relatively low due to the short length of texts in the S records, providing less context to judge the impact on patients’ daily lives, especially for the AE-L model (the mean word number of the S records was 38.8 [SD 29.4; Table 1], similar to the sentence-level tasks in our previous work [42,43]). However, we consider a true adverse event signal proportion of more than 80% in this study represents a promising outcome, as this is the first attempt to apply our deep learning models to a different source of patients’ concern data, and the extracted positive cases would be worthy of evaluation by a medical professional, as the potential adverse events could be caused by drugs taken by the patients.

When the deep learning models were applied to DIPEx-Japan interview transcripts, including patients’ concerns, the proportion of true adverse event signals was also more than 80% (for All AE: n=73, 86.9% and for HFS and AE-L: n=18, 100%). The difference in the results between pharmaceutical care S records and DIPEx-Japan interview transcripts was the features of false-positives, descriptions about lack of symptoms or confirmation of improving condition in S records versus explanations about disease or its prognosis, stories about when their cancer was discovered, or emotional changes in interview transcripts. This is considered due to the difference in the nature of the data source; the pharmaceutical care records were generated in a real-time manner by pharmacists through their daily work, where adverse event signals are proactively monitored, while the interview transcripts were purely based on patients’ retrospective memories. Our deep learning models were able to extract true adverse event signals with an accuracy of more than 80% from both text data sources in spite of the difference in their nature. When looking at future implementation of the deep learning models in society (discussed in the Potential for Deep Learning Model Implementation in Society section), it may be desirable to further adjust deep learning models to reduce false-positives depending upon the features of the data source.

### Identification of Important Adverse Events Requiring Medical Intervention

To assess whether the models could extract clinically important adverse event signals, we investigated interventions by health care professionals connected with the adverse event signals that are identified by our deep learning models. In the 200 randomly extracted S records, only 26 (13%) consisted of adverse event signals, leading to any intervention by health care professionals. On the other hand, the proportion of signals associated with interventions was increased to 107 (64.1%) and 91 (46.4%) in the S records extracted as positive by the HFS and AE-L models, respectively (Figures 1 and 2). These results suggest that both deep learning models can screen clinically important adverse event signals that require intervention from health care professionals. The performance level in screening adverse event signals requiring medical intervention was higher in the HFS model than in the AE-L model (n=107, 64.1% vs n=91, 46.4%; Figures 1 and 2). Since the target events were specific and adverse event signals of HFS were narrowly defined, which is one of the typical side effects of some anticancer drugs, we consider that health care providers paid special attention to HFS-related signals and took action proactively. In both deep learning models, similar trends were observed in actions taken by health care professionals in response to extracted adverse event signals; common actions were attempts to manage adverse event symptoms by symptomatic treatment or other mild interventions, including educational guidance from pharmacists or recommendations for patients to visit a doctor. More direct interventions focused on the causative drugs (ie, “dose reduction or discontinuation of anticancer treatment”) amounted to less than 5%; 7 (4.2%) for the HFS model and 6 (3.1%) for the AE-L model (Figures 1 and 2). Thus, it appears that our deep learning models can contribute to screening mild to moderate adverse event signals that require preventive actions such as symptomatic treatments or professional advice from health care providers, especially for patients with less sensitivity to adverse event signals or who have few opportunities to visit clinics and pharmacies.

### Ability to Catch Real Side Effect Signals of Anticancer Drugs

Based on the drug prescription history associated with S records extracted as HFS or AE-L positive, the type and duration of anticancer drugs taken by patients experiencing the adverse event signals were investigated. For the HFS model, the most common MoA of anticancer drug was antimetabolite (fluoropyrimidine: n=59, 38.8%), followed by kinase inhibitors (n=49, 32.2%, of which EGFR inhibitors and multikinase inhibitors accounted for n=28, 18.4% and n=14, 9.2%, respectively) and aromatase inhibitors (n=24, 15.8%; Table 3). It is known that fluoropyrimidine and multikinase inhibitors are typical HFS-inducing drugs [55-58], suggesting that the HFS model accurately extracted HFS side effect signals derived from these drugs. Note that symptoms such as acneiform rash, xerosis, eczema, paronychia, changes in the nails, arthralgia, or stiffness of limb joints, which are common side effects of EGFR inhibitors or aromatase inhibitors [59,60], might be extracted as closely related expressions to those of HFS signals. When looking at the MoA of anticancer drugs for patients with adverse event signals identified by the AE-L model, antimetabolite (fluoropyrimidine) was the most common one (n=53, 33.8%), as in the case of those identified by the HFS model, followed by kinase inhibitors (n=31, 19.7%) and antiandrogens (n=27, 17.2%; Table 5). Since the AE-L model targets a broad range of adverse event symptoms, it is difficult to rationalize the relationship between the adverse event signals and types of anticancer drugs. However, the type of anticancer drugs would presumably closely correspond to the standard treatments of the cancer types of the patients. Based on the prescribed anticancer drugs, we can infer that a large percentage of the patients had breast or lung cancer, indicating that our study results were based on data from such a population. Thus, a possible direction for the expansion of this research would be adjusting the deep learning models by additional training with expressions for typical side effects associated with standard treatments of other cancer types. To interpret these results correctly, it should be noted that we could not investigate anticancer treatments conducted outside of the pharmacies (eg, the time-course relationship with intravenously administered drugs would be missed, as the administration will be done at hospitals). To further evaluate how useful this model is in side effect signal monitoring for patients with cancer, comprehensive medical information for the eligible patients would be required.

### Suitability of the Deep Learning Models for Specific Adverse Event Symptoms

Among the adverse event signals identified by the AE-L model, the type of symptom was categorized according to a predefined annotation guideline that we previously developed [43]. The most frequently recorded adverse event signals identified by the AE-L model were “pain or numbness” (n=57, 36.3%), “fever” (n=46, 29.3%), and “nausea” (n=40, 25.5%; Table 6). Since the pharmaceutical care records had information about interventions by health care professionals, the frequency of the presence or absence of the interventions for each symptom was examined. A trend toward a greater proportion of interventions was observed in “fever,” “nausea,” “diarrhea,” “constipation,” “vomiting,” and “edema” (Figure 3, black boxes). There seem to be 2 possible explanations for this: these symptoms are of high importance and require early medical intervention or effective symptomatic treatments are available for these symptoms in clinical practice so that medical intervention is an easy option. On the other hand, a trend for a smaller proportion of adverse event signals to result in interventions was observed for “pain or numbness,” “fatigue,” “appetite loss,” “rash or itchy,” “taste disorder,” and “dizziness” (Figure 3, gray boxes). The reason for this may be the lack of effective symptomatic treatments or the difficulty of judging whether the severity of these symptoms justifies medical intervention by health care providers. In either case, there may be room for improvement in the quality of medical care for these symptoms. We expect that our research will contribute to a quality improvement in safety monitoring in clinical practice by supporting adverse event signal detection in a cost-effective manner.

### Potential for Deep Learning Model Implementation in Society

Although we evaluated our deep learning models using pharmaceutical care records in this study, the main target of future implementation of our deep learning models in society would be narrative texts that patients directly write to record their daily experiences. For example, the application of these deep learning models to electronic media where patients record their daily experiences in their lives with disease (eg, health care–related e-communities and disease diary applications) could enable information about adverse event signal onset that patients experience to be provided to health care providers in a timely manner. Adverse event signals can automatically be identified and shared with health care providers based on the concern texts that patients post to any platform. This system will have the advantage that health care providers can efficiently grasp safety-related events that patients experience outside of clinic visits so that they can conduct more focused or personalized interactions with patients at their clinic visits. However, consideration should be given to avoid an excessive burden on health care providers. For instance, limiting the sharing of adverse event signals to those of high severity or summarizing adverse event signals over a week rather than sharing each one in a real-time manner may be reasonable approaches for medical staff. We also need to think about how to encourage patients to record their daily experiences using electronic tools. Not only technical progress and support but also the establishment of an ecosystem where both patients and medical staff can feel benefit will be required. Prospective studies with deep learning models to follow up patients in the long term and evaluate outcomes will be needed. We primarily looked at patient-authored texts as targets of implementation, but our deep learning models may also be worth using medical data including patients’ subjective concerns, such as pharmaceutical care S records. As this study confirmed that our deep learning models are applicable to patients’ concern texts tracked by pharmacists, it should be possible to use them to analyze other “patient voice-like” medical text data that have not been actively investigated so far.

### Limitations

First, the major limitation of this study was that we were not able to collect complete medical information of the patients. Although we designed this study to analyze patients’ concerns extracted by the deep learning models and their relationship with medical information contained in the pharmaceutical care records, some information could not be tracked (eg, missing history of medical interventions or anticancer treatment at hospitals as well as diagnosis of patients’ primary cancers). Second, there might be a data creation bias in S records for patients’ concerns by pharmacists. For example, symptoms that have little impact on intervention decisions might less likely be recorded by them. It should be also noted that the characteristics of S records may not be consistent at different community pharmacies.

### Conclusions

Our deep learning models were able to screen clinically important adverse event signals that require intervention by health care professionals from patients’ concerns in pharmaceutical care records. Thus, these models have the potential to support real-time adverse event monitoring of individual patients taking anticancer treatments in an efficient manner. We also confirmed that these deep learning models constructed based on patient-authored texts could be applied to patients’ subjective information recorded by pharmacists through their daily work. Further research may help to expand the applicability of the deep learning models for implementation in society or for analysis of data on patients’ concerns accumulated in professional records at pharmacies or hospitals.

## Appendix

Multimedia Appendix 1 Performance evaluation of deep learning models.

Multimedia Appendix 2 Examples of S records and sample interview transcripts.

## Abbreviations

AE-L: adverse events limiting patients’ daily lives
All AE: all or any adverse event
BERT: Bidirectional Encoder Representations From Transformers
DIPEx-Japan: Database of Individual Patient Experiences-Japan
EGFR: epidermal growth factor receptor
HFS: hand-foot syndrome
MoA: mechanism of action
PRO: patient-reported outcome
SOAP: subjective, objective, assessment, plan

## Data Availability

The data sets generated and analyzed during this study are available from the corresponding author on reasonable request.
