# Supplementary material for: Adverse Event Signal Detection Using Patients’ Concerns in Pharmaceutical Care Records: Evaluation of Deep Learning Models
Source: J Med Internet Res. 2024 Apr 16;26:e55794. doi: 10.2196/55794 (PMC11061790; doi:10.2196/55794)
Supplement: Multimedia Appendix 1 [file jmir_v26i1e55794_app1.docx]

## Method

From the original data source of pharmaceutical care records, 1000 randomly extracted S records were used to check the performance of our deep learning models (HFS, All AE, and AE-L) in this study. The 1,000 S records were manually annotated as HFS positive or not, All AE or not, or AE-L or not by annotators (SN, YY and KS) according to the same annotation guidelines and process that we implemented in our previous work [42,43]. The performance of each model was evaluated in terms of precision, recall and F_1_ score (see below), which are commonly used for evaluation of binary classification tasks.

$$Precision=\frac{\# of True Positive}{\# of True Positive+False Positive}$$

$$Recall= \frac{\# of True Positive}{\# of True Positive+False Negative}$$

$$F_{1} score=\frac{2*precision*recall}{precision+recall}$$

## Results

The performance results when our deep learning models were applied to the 1,000 S records are summarized in Table S1 below. There were 20 (2.0%), 297 (29.7%), and 6 (0.6%) true positives for HFS, ALL AE and AE-L, respectively (TP + FN in Table S1). For reference, the performance scores of the deep learning models we observed at sentence level tasks in our pre-work, where we used blog posts written by cancer patients as the data source, are also listed in Table S2 below.

**Table S1.** Performance levels and prediction results for S records in this study.

|  | Precision | Recall | F_1_ score | TP | FN | FP | TN |
| --- | --- | --- | --- | --- | --- | --- | --- |
| HFS | 0.88 | 0.35 | 0.50 | 7 | 13 | 1 | 979 |
| All AE | 0.66 | 0.62 | 0.64 | 185 | 112 | 95 | 608 |
| AE-L | 0.17 | 0.33 | 0.22 | 2 | 4 | 10 | 984 |

TP: True Positive, FP: False Positive, FN: False Negative, TN: True Negative

**Table S2.** Performance levels for sentence tasks in our pre-work.

|  | Precision | Recall | F_1_ score |
| --- | --- | --- | --- |
| HFS [42] | 0.45 | 0.66 | 0.54 |
| All AE* | 0.71 | 0.35 | 0.46 |
| AE-L* | 0.53 | 0.14 | 0.22 |

* The performance scores of the T5 model at the sentence level tasks are newly shown here as they were not included in the published pre-work [43]
