# Supplementary material for: Adverse Event Signal Detection Using Patients’ Concerns in Pharmaceutical Care Records: Evaluation of Deep Learning Models
Source: J Med Internet Res. 2024 Apr 16;26:e55794. doi: 10.2196/55794 (PMC11061790; doi:10.2196/55794)
Supplement: Multimedia Appendix 2 [file jmir_v26i1e55794_app2.docx]

**Table S1.** Examples of actual S records among HFS-positive cases.

| **Original in Japanese** | **English translation** |
| --- | --- |
| 昨日左足が痛いと訴えがあり、ふらつきもあります。 | Yesterday, suffered from pain in the left leg and also wobbliness. |
| 爪の方は、まだ赤みと痛みがあります。軟膏は使っていますよ。 | The nail is still red and sore. Using ointment. |
| 味覚がなんだか違和感あります。指先もヒリヒリする。 | My sense of taste is somewhat strange. My fingertips also feel HIRI-HIRI* (tingling). |
| 今回から後発で服用してみようと思う。抗がん剤を服用するとどうしても指の先が黒くなって、関節の裏が切れてしまう。目は眩しいと涙が溢れて白っぽくなることもある。副作用だから仕方ないんだよね。顔、爪、手、足などが黒ずんできた | I'm going to try a generic drug from now on. When I take an anticancer drug, my fingertips inevitably turn black and the backs of my joints get cut. My eyes sometimes turn white from dazzling and tears. They are side effects, so there's nothing I can do about it. My face, nails, hands, feet, etc. have darkened. |
| エスワンタイホウは休薬です。手指、掌の黒ずみあり。口内炎は出来ていないが口の周りがピリピリする感じあり | Stopped S-1 TAIHO**. Darkening of fingers and palms is present. No stomatitis, but there is sensation of PIRI-PIRI* (tingling) around my mouth. |

* Onomatopoeic expression in Japanese, ** Name of anticancer drug

**Table S2.** DIPEx-Japan interview transcript extracted as HFS-positive by the HFS model (only one [0.2%] in 508 transcripts).

| **Original in Japanese** | **English translation** |
| --- | --- |
| 4クール目に入って、「あと4回で治療が終わる！」とちょっと気持ちも上がってきたころに、爪に症状が出てきて、爪が真っ黒く変色したりとか、あと、爪に少しでも物が当たると激痛が走る感じで、もう足も手ももう物が触れると本当に激痛が走るのと、あとは出血してきたりとかするので、爪がはがれてしまったりとかしていて、足がもうどうしようもなかったんですけれど、手のほうはガーゼみたいなもので保護、爪を保護したりとかしていました。で、もう指の感覚とかが感覚異常で出ていて、さらに爪も出ていたので、もう手使うことがもう嫌なのと、足ももう動かしたくない、みたいな感じで、すごく動くことに恐怖心が出てきました。 | I was in the fourth course, and when I was thinking "I'm going to finish the treatment in four sessions!”, symptoms appeared on my nails, like a black discoloration of my nails. In addition, the pain was so intense that my feet and hands would start to bleed when they hit things, and my nails were peeling off. I couldn't do anything about my feet, but I was able to use gauze to protect my hands and nails. I was already experiencing abnormal sensation in my fingers, as well as my nails, so I didn't want to use my hands anymore, and I didn't want to move my feet anymore either. I became so scared to move. |

**Table S3.** Examples of actual S records among AE-L-positive cases.

| **Original in Japanese** | **English translation** |
| --- | --- |
| 毎日3回飲んでいると思います。前にもらったのがあと少し残っているので出してもらっています。仕事も行ってはいたけど、、、って感じです。胸水で息が苦しい、できない状態です。少しだけしか歩けません。それと、右脇腹の痛みがあって最近眠れないんです。 | I think I take it three times daily. I have a few more left from the last one I got, so I am taking it. I had been going to work, but now I can't.... I am having a hard time breathing, can't do it because of the pleural effusion. I cannot walk for long. Also, I have pain in my right side that prevents my sleep these days. |
| 1番辛いSEは腹痛ですね。アセトアミノフェン追加になってから少しは良くなってきているんだけど。アドは足の裏の皮が剥がれてきています。痛みとかはでていません。急激な腹痛やさしこむような激しい腹痛がある→2mgに減量 | The hardest side effect is stomach ache. It's getting a little better since the acetaminophen was added though. And the skin on the bottom of my feet is peeling off. No pain. Sudden stomach ache or piercing severe abdominal pain -> dose reduced to 2 mg. |
| 現在痛みなく落ち着いています。食事も取れているしだるさもない状態です。熱は39℃くらい出ています。炎症反応出ているので抗生剤出ました。血圧は100/79で安定している。下痢はあります。眠気や便秘はないです。 | It's currently pain-free and calm. I'm able to eat and am not lethargic. My body temperature is around 39 degrees Celsius. Antibiotics were given due to inflammatory reaction. Blood pressure is stable at 100/79. There is diarrhea but no drowsiness or constipation. |
| 月曜日に抗がん剤治療しましたが、昨日の昼に悪心の症状があり10回くらい嘔吐しました。偶数日に抗がん剤治療するので翌朝にナゼア服用するように指示ありました。 | I was treated with anticancer drugs on Monday, but last afternoon I had nausea and vomited about 10 times. I was instructed to take Nasea* the next morning since I will be treated with the anticancer drugs on even days. |
| 抗がん剤治療実施。前回のクールで微熱と吐き気が１週間くらい続いてしまって何もしたくない感じでした。食べ物は果物や汁物などをなんとか食べられた。下痢はしてない。下剤も調節している。 | Anticancer drug treatment was implemented. I had a slight fever and nausea for about a week after the last treatment and didn't want to do anything. I managed to eat light food, like fruits and juices. No diarrhea. Laxatives have been adjusted. |

* Name of an antiemetic 5-HT3 antagonist drug

**Table S4.** DIPEx-Japan interview transcripts extracted as positive by the All AE or AE-L model.

| Item | All AE-positive | AE-L-positive |
| --- | --- | --- |
| 1. Number of positive (% in N = 508) | 84 transcripts (16.5%) | 18 transcripts (3.5%) |
| 1. Adverse event signal (% in a)  - Yes - No (ie, false positive) - Explanation about disease or its prognosis - Story when their cancer was discovered - Change in emotional condition | - 73 transcripts (86.9%) - 11 transcripts (13.1%)   - 6 transcripts (7.1%)   - 4 transcripts (4.8%)   - 1 transcript (1.2%) | - 18 transcripts (100.0%) - 0 transcripts (0.0%)   - N/A   - N/A   - N/A |

**Table S5.** Examples of DIPEx-Japan interview transcripts extracted as positive by the All AE (a) or AE-L model (b).

| 1. **An example of All AE-positive** | |
| --- | --- |
| **Original in Japanese** | **English translation** |
| それから、まあ、子育てをしながら、治療を続けて、で、治療が終わって、なったんですけど。やっぱり、なかなか、その体が100%戻るっていうのには、時間がかかって、自分の場合は、あの、リンパを結構取っているので、あの、腕がですね、よく腫れるんですね。リンパ浮腫まで、すごく腫れるっていうことはないんですけど、少し疲れたり、あと、ま、手の先を怪我してばい菌が入ったりすると、腕にあるリンパがすごく赤くリンパのふさふさに沿って斑点みたいに赤くなって、で、あの、熱が出るんですね。で、ま、無理したときには、必ずそれが出てしまうんですけど。で、まあ、必ず乳腺の先生のところに行って、自分の場合、抗生剤を飲んだら治るので、で、じゃ、お薬で治してっていうの繰り返して。そういうのもあって、やっぱり、無理はできないなあというのを感じながら、まあ、生活をして。 | While raising my child, I continued the treatment, and finally the treatment was over. It took a long time to get back to 100% condition. In my case, my arms often swelled up because I had a lot of lymph nodes taken out. It doesn't swell up like lymphedema, but when I was a little tired, or if I injured the tip of my hand and germs got into it, my arm developed red spots along the lymphatic follicles, and I got a fever. Whenever I overworked, it would appear. Then, I would always go to a doctor in the breast department, and in my case, it would go away after I took antibiotics, so I would repeat the process of treating it with medicine. So, I figured out I couldn’t take on too much, and kept a quiet daily life. |

**Table S5.** (continued)

| 1. **An example of AE-L-positive** | |
| --- | --- |
| **Original in Japanese** | **English translation** |
| 抗がん剤でつらかったのは、やはり強い薬からやり始めるそうなので、私は薬に大変敏感なほうらしいんです。それで、やり始めて8時間たつと、もう体にサインが来るんですね。吐き気とか熱とかっていう、まあ、熱は出ましたけれども、嘔吐とかっていう苦しみはないんですが、何ともいえないもうろうとしてきて。ティッシュペーパーの箱の周りに、レースのカバーをかけてたんですが、そのレースが波を打っているように見えたり、そのレースの縁取りに虫がはっているように見えたり、そういう状態が続き、それがとっても怖くて、もう鳥肌が立つぐらい、怖くて怖くてしょうがなかったことを思い出します。あとは、抗がん剤やってたとき、とってもつらかったのは、3日間だけなんですね。3日間だけ丸まって、それこそ赤ちゃんがお母さんのおなかの中にいる、羊水にいるような形で丸まって、トイレに行くだけ、行くだけしか、動けない。それすらも苦痛なんですけど、行かないわけにはいかないから用を足しにいく。そしたら、また帰ってきて、もうとにかく眠り姫っていわれるぐらい、もう寝てばっかりいるんですけど、寝てはいないんです。眠っているように見えるんだけど、みんなの動き、全部解っているんです。ものすごい疲れなんです。 | What was hard about the anticancer drugs was that they started with the strongest drugs, and I was apparently very sensitive to the drugs. After 8 hours of treatment, my body would start to show signs of nausea and fever. I had a fever, but I did not suffer from vomiting or anything like that. I was getting lightheaded. I had a box of tissue paper with a lace cover around it, and the lace looked like it was waving to me, and the edges of the lace looked like they were covered with insects, and I was so scared that I had goosebumps. I remember that I was really scared by such experiences. The other thing that was very painful to me when I was on anticancer drugs was when I had to curl up for three days, like a baby in its mother's tummy, curling up in amniotic fluid, and could only move to go to the bathroom. Even that was painful, but I had to go, so I went to relieve myself. Then I would come back again, and I would be like "Sleeping Beauty," and I would sleep all the time. Although I wasn't sleeping actually. I looked like I was asleep, but I understood everything that everyone was doing. I was extremely tired. |
